# Egr1 plays a major role in the transcriptional response of white adipocytes to insulin and environmental cues

**DOI:** 10.3389/fcell.2022.1003030

**Published:** 2022-09-28

**Authors:** A. B. Meriin, N. Zaarur, D. Roy, K. V. Kandror

**Affiliations:** ^1^ Department of Biochemistry, Boston University School of Medicine, Boston, MA, United States; ^2^ Department of Neuroscience, The Ohio State University, Columbus, OH, United States

**Keywords:** Egr1, insulin, adipocytes, ATGL, leptin, circadian rhythms

## Abstract

It is believed that insulin regulates metabolic functions of white adipose tissue primarily at the post-translational level *via* the PI3K-Akt-mediated pathway. Still, changes in transcription also play an important role in the response of white adipocytes to insulin and environmental signals. One transcription factor that is dramatically and rapidly induced in adipocytes by insulin and nutrients is called Early Growth Response 1, or Egr1. Among other functions, it directly binds to promoters of leptin and ATGL stimulating the former and inhibiting the latter. Furthermore, expression of Egr1 in adipocytes demonstrates cell autonomous circadian pattern suggesting that Egr1 not only mediates the effect of insulin and nutrients on lipolysis and leptin production but also, coordinates insulin action with endogenous circadian rhythms of adipose tissue.

## Introduction

White adipose tissue (WAT) plays a key role in mammalian physiology and pathophysiology (Sakers et al., 2022). There are at least three highly important metabolic functions in the body that are attributed primarily (but not exclusively) to WAT: dynamic storage of triglycerides (TG), secretion of adipokines, such as leptin, and regulated glucose uptake. Each of these functions is controlled by insulin at the level of transcription and translation as well as by post-translational mechanisms (Kandror, 2015; Grabner et al., 2021). Although glucose transporter four mediated glucose uptake is believed to be up-regulated by insulin exclusively at a post-translational level (Calejman et al., 2022), preservation of the low level of glucose uptake in basal adipocytes not treated with insulin requires continuous RNA- and protein biosynthesis *de novo* (Meriin et al., in press). The details of the transcriptional control of glucose homeostasis in adipocytes are not yet known; however, it has been established that effects of insulin on lipolysis and leptin expression are mediated at least in part, by Early Growth Response transcription factor, Egr1.

Egr1 (a.k.a. NGFI-A, Zif268, TIS8, and Krox24) is a zinc finger transcription factor that belongs to the family of primary response genes (Fowler et al., 2011). Like other members of this family, Egr1 participates in growth control, differentiation, and cancer progression (Thiel et al., 2010; Pagel and Deindl, 2011; Wang et al., 2021). The role of Egr1 in regulation of metabolism remains poorly explored and will be the focus of this review.

### Insulin and nutrients rapidly but transiently induce Egr1 in adipocytes

Basal adipocytes maintain low, almost undetectable levels of Egr1. Treatment of adipocytes with insulin causes a dramatic induction of both Egr1 mRNA (Alexander-Bridges et al., 1992; Sartipy and Loskutoff, 2003) and protein (Chakrabarti et al., 2013; Mohtar et al., 2019) both *in vivo* and *in vitro*. Incubation of cultured adipocytes with glucose (Supplementary Image S1) or high fat feeding of mice *in vivo* (Chakrabarti et al., 2013) also elevate expression of Egr1.

Similar to other primary response genes (Fowler et al., 2011)**,** expression of Egr1 in various cell types is regulated at the level of transcription (Thiel and Cibelli, 2002; Thiel et al., 2010; Pagel and Deindl, 2011). In adipocytes, the effect of insulin on the Egr1 mRNA is mediated by Erk (Singh et al., 2015). Interestingly, this increase in Egr1 mRNA contributes relatively little to insulin-triggered up-regulation of the Egr1 protein in adipocytes. The latter takes place primarily at the level of translation *via* the mTORC1-4E-BP-mediated axis (Singh et al., 2015) and depends on the highly structured 5′-UTR of the Egr1 mRNA. To this end, we have deleted the 5′-UTR of the Egr1 mRNA using the CRISPR/Cas9 technique. This procedure alone brings up expression of the Egr1 protein to the maximum, so that insulin does not have any additional stimulatory effect (Mohtar et al., 2019).

Expression of the Egr1 protein reaches its maximum after 1 h of insulin stimulation and goes back to the basal level after approximately 4 h (Mohtar et al., 2019). Still, insulin does not significantly stimulate degradation of Egr1 (Supplementary Figure S2) and its rapid decline is most likely explained by inherent instability of the Egr1 mRNA (Singh et al., 2015) and protein.

### Egr1 directly regulates expression of adipose triglyceride lipase and lipolysis in adipocytes

In mammalian organism, most energy is stored in adipose tissue in a form of TG in distinct intracellular organelles called lipid droplets (LDs). Upon TG hydrolysis, FA are circulated in the blood to cells and tissues where they are taken up and used for energy production and synthesis of complex lipids. Despite their fundamental physiological importance, an oversupply of FA is highly detrimental as it causes abnormal lipid partitioning and lipotoxicity which in turn, impairs membrane function, induces ER stress, mitochondrial dysfunction, inflammation, cell death, insulin resistance, and other metabolic disease (Unger et al., 2010). The fine balance between healthy and unhealthy levels of circulating FA is maintained *via* a tight control of lipolysis coordinated with food intake. Thus, an increase in circulating levels of FA after food intake is normally compensated by insulin-mediated suppression of lipolysis in adipose tissue. This may be crucial for at least two reasons. First, dietary FAs in combination with those FAs produced endogenously by lipolysis may overcome all existing defense mechanisms of the body and impose a significant nutritional stress on cells and tissues leading to lipotoxicity and metabolic disease. Second, arrest of lipolysis when nutrients are abundant protects valuable fat reserves from unnecessary depletion. Failure of insulin to restrain lipolysis is a serious metabolic defect that leads to T2D and other health problems (McGarry, 1992; McGarry, 2002).

Complete lipolysis, i.e., hydrolysis of TG to glycerol and FA, is performed jointly by tri-, di-, and monoacylglyceride lipases (Grabner et al., 2021). The rate-limiting lipolytic enzyme, ATGL, is responsible for the bulk of triacylglycerol hydrolase activity in various cells. In other words, in every experimental model tested thus far, elevated ATGL expression increases, while attenuated ATGL expression decreases, both basal and cAMP-stimulated lipolysis (Jenkins et al., 2004; Villena et al., 2004; Zimmermann et al., 2004; Gronke et al., 2005; Haemmerle et al., 2006; Kershaw et al., 2006; Kurat et al., 2006; Smirnova et al., 2006; Miyoshi et al., 2008; Bezaire et al., 2009; Chakrabarti et al., 2010). ATGL has low affinity for di- and monoacylglycerides (Grabner et al., 2021). The major diacylglyceride lipase in adipocytes is hormone-sensitive lipase, or HSL and monoacylglyceride products of HSL are hydrolyzed by monoacylglyceride lipase (Grabner et al., 2021).

According to current views, lipolysis is regulated by catecholamines primarily at the post-translational level with the cAMP/cGMP-mediated signaling pathways playing the key role in this process. Briefly, phosphorylation of the lipid droplet protein perilipin and HSL by PKA and/or PKG leads to the recruitment of HSL to lipid droplets and activation of the enzyme. At the same time, a protein co-factor of ATGL, Abhd5 (a.k.a. CGI-58) dissociates from phosphorylated perilipin and activates ATGL (Lafontan and Langin, 2009; Grabner et al., 2021). Jointly, both processes rapidly and significantly stimulate lipolysis. On the contrary, insulin inhibits lipolysis and promotes accumulation of TG. Within this model, the effect of insulin is attributed primarily to the inhibition of cAMP-mediated signaling *via* Akt-dependent (Kitamura et al., 1999; Duncan et al., 2007) and independent (Choi et al., 2010) mechanisms.

In addition, regulation of lipolysis *in vivo* by such physiological stimuli as feeding, fasting, hypoxia, and physical exercise is accompanied and likely mediated by changes in the ATGL expression (Fortier et al., 2004; Villena et al., 2004; Lake et al., 2005; Kershaw et al., 2006; Kim et al., 2006; Alsted et al., 2009; Nielsen et al., 2011; Han et al., 2019). In particular, Supplementary Figure S3 shows that insulin rapidly and completely shuts ATGL transcription in cultured adipocytes. Expression of the ATGL protein follows the levels of cognate mRNA suggesting that expression of ATGL is controlled primarily at the level of transcription.

Thus, not only post-translational regulation of the enzymatic activity but also, precise control of the ATGL transcription defines the rates of lipolysis and FA homeostasis. However, unlike post-translational regulation that has been studied in much detail, little has been known about regulation of ATGL expression.

To this end, we have initiated a search for the pathways that regulate transcription of ATGL by nutrients and insulin. We have found two pathways: the Egr1-mediated pathway that inhibits lipolysis by decreasing transcription of ATGL (Chakrabarti et al., 2010; Chakrabarti et al., 2013; Singh et al., 2014) and the Sirt1/FoxO1-mediated pathway that activates lipolysis by increasing transcription of ATGL (Chakrabarti and Kandror, 2009; Chakrabarti et al., 2011; Jung et al., 2019). Both Egr1 (but not its close relative, Egr2) and FoxO1 directly bind to the ATGL promoter with different outcomes: Egr1 inhibits while FoxO1 stimulates its activity (Chakrabarti and Kandror, 2009; Chakrabarti et al., 2013; Singh et al., 2015) leading to corresponding changes in the ATGL expression and lipolysis. Importantly, regulation of ATGL expression by Egr1 is conserved in evolution from yeast to mammals and thus should be essential for metabolic control (Chakrabarti et al., 2013).

Expression of ATGL can be regulated by other transcription factors as well. Thus, early experiments have demonstrated that expression of ATGL is stimulated by PPARγ (Kim et al., 2006; Kershaw et al., 2007). Furthermore, interferon regulatory factor 4 induced in adipocytes by starvation *via* FoxO1 up-regulates transcription of ATGL (Eguchi et al., 2011), while insulin-induced transcription factor Snail1 suppresses its transcription (Sun et al., 2016).

Apparently, transcriptional control of lipolysis works on a different time scale, than the previously established mechanism of the short-term insulin action by inhibition of cAMP-mediated signaling to HSL and perilipin. The first one takes 4–6 h while the latter occurs within minutes. Both types of regulation seem essential for the physiological control of circulating FA.

### Egr1 regulates leptin expression in adipocytes

Leptin, a 16 kDa product of the *ob* gene (Zhang et al., 1994), is synthesized predominantly in adipocytes and targets the central nervous system. It has been established as a major metabolic regulator that controls food intake, energy expenditure, neuroendocrine functions, carbohydrate and lipid metabolism, and several other important physiological functions of the mammalian organism (Ahima and Flier, 2000; Friedman, 2009; Dalamaga et al., 2013; Zeng et al., 2015). The discovery of leptin over two decades ago has completely changed the landscape of metabolic research and opened a new era in obesity studies.

Regardless of how leptin exerts its biological activity, it is essential that leptin production in adipocytes is coupled to nutrient uptake and energy status of the body. As circulating leptin and insulin levels increase after feeding and decrease after food deprivation (Frederich et al., 1995; Levy et al., 1997; Ahima and Flier, 2000), the predominant hypothesis in the field has been that leptin expression is controlled by insulin. Indeed, multiple studies have shown that insulin increases leptin production by adipose cells both *in vivo* and *in vitro* (Ahima and Flier, 2000). Although this regulatory connection is central to all proposed mechanisms of leptin action, its mechanism has remained unknown. Recently, we have found that insulin and nutrients activate leptin transcription in adipocytes *via* the same mTORC1-Egr1 axis that plays the central role in downregulation of ATGL (Mohtar et al., 2019). Very briefly, Egr1 directly interacts not only with the ATGL promoter (see above) but also, with the leptin promoter, suppressing the former and activating the latter. This mechanism may explain the long-known connection between food intake and circulating leptin (Frederich et al., 1995; Levy et al., 1997; Ahima and Flier, 2000).

Multiple lines of evidence demonstrate that insulin and nutrients control expression of leptin not exclusively at the level of transcription but also, at the level of translation, secretion, and even degradation (Lee and Fried, 2006; Lee and Fried, 2009; Kandror, 2015). It has been shown that mTORC1 plays a major role in the translation control of leptin (Roh et al., 2003; Lee and Fried, 2006), but its input into leptin secretion and degradation has not yet been studied.

In any case, a reverse regulation of ATGL and leptin by the same mTORC1-Egr1 axis may help to coordinate and even to synchronize changes in lipolysis (*via* ATGL) with food intake and energy expenditure (*via* leptin). Counter-regulation of leptin production and lipolysis may be maintained by other mechanisms as well. Thus, it has been demonstrated that hypoleptinemia may activate hypothalamic-pituitary-adrenal axis to promote lipolysis in fat (Perry et al., 2018).

### Other metabolic effects of Egr1

Two polymorphisms in Egr1 have been associated with impaired lipid metabolism in humans (Brand et al., 2000), and several recent reports have confirmed that Egr1 is intimately involved in the regulation of lipid metabolism. Thus, in addition to the regulation of ATGL and leptin (Chakrabarti et al., 2013; Mohtar et al., 2019), Egr1 has been implicated in adipogenesis (Boyle et al., 2009) and browning of white adipocytes (Milet et al., 2017). Interestingly, Egr1 has a negative effect on adipose differentiation, while Egr2 is pro-adipogenic (Boyle et al., 2009). It has also been reported that Egr1 regulates insulin biosynthesis (Muller et al., 2012) and resistance (Shen et al., 2011), and cholesterol biosynthesis (Gokey et al., 2011). A comprehensive and balanced picture of all metabolic effects of Egr1 and Egr2 has yet to be established.

### A role of Egr1 in the circadian regulation of ATGL and leptin

**FIGURE 1:**
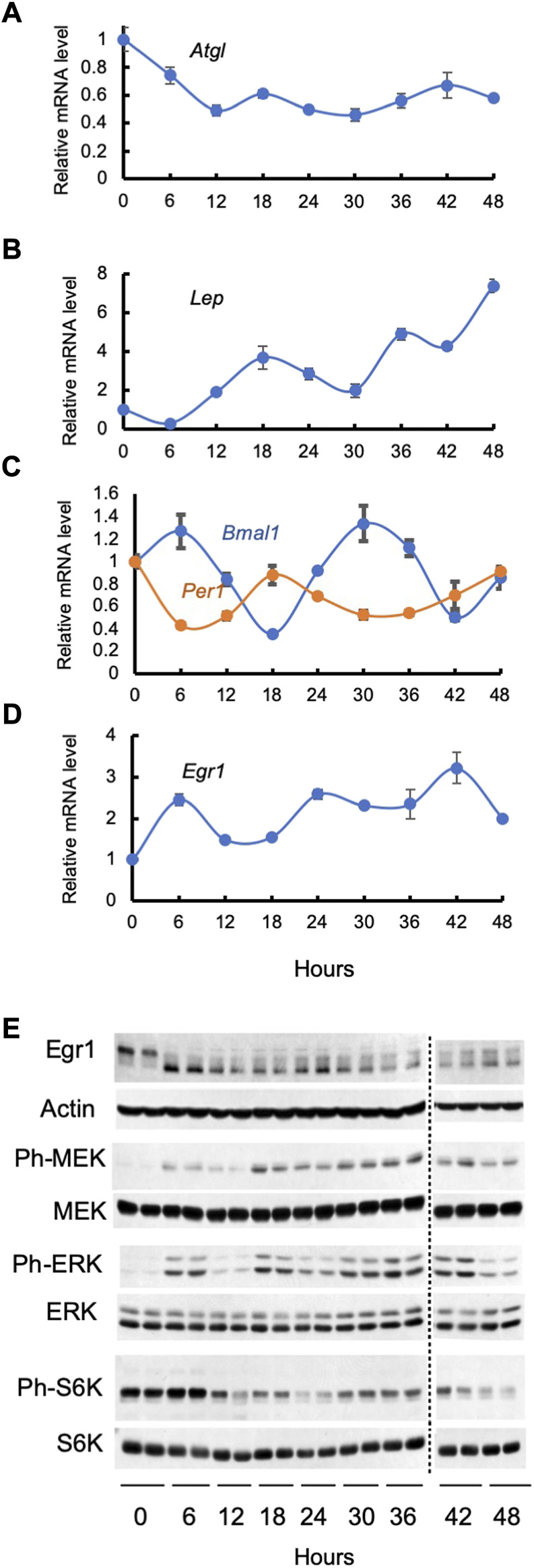
Cell autonomous oscillations in synchronized 3T3-L1 adipocytes. Cells were serum starved for 2 h, treated with 50% horse serum for 2 h, washed, and monitored for the next 48 h under normal culturing conditions without serum. Panels **(A–D)**: levels of various mRNAs were determined by qPCR data in three independent experiments; mean values ± SE are shown. Panel **(E)**: lysates of two biological replicates were analyzed by Western blotting; both are shown. The following antibodies from Cell Signaling Technology have been used: rabbit monoclonal antibody against Egr1 (Catalog #4153), mouse monoclonal antibody against *β*-Actin (Catalog #3700), rabbit monoclonal antibody against phospho-MEK1/2 (Catalog #9154), rabbit monoclonal antibody against MEK1/2 (Catalog #8727), rabbit monoclonal antibody against phospho-Erk1/2 (Catalog #4370), rabbit monoclonal antibody against Erk1/2 (Catalog # 4695), rabbit monoclonal antibody against phospho-S6 Kinase (Catalog # 9208), rabbit monoclonal antibody against S6 Kinase (Catalog # 2708).

Circadian patterns of circulating free fatty acids in humans have been known for a long time (Schlierf and Raetzer, 1972); more recently, they have been attributed to oscillations of ATGL- and less so, HSL-mediated lipolysis in white adipose tissue (Shostak et al., 2013b). It has been also shown that in both diurnal (humans, monkeys) and nocturnal (rats, mice) animals food intake is regulated by circadian changes in plasma leptin levels (reviewed in (Froy and Garaulet, 2018). In line with these experiments, we have found that expression of ATGL and leptin oscillates in synchronized adipocytes cultured in serum-free media in the absence of any putative light or food entrainable oscillator (Figures 1A,B).

In diverse organisms, the circadian clock coordinates metabolism with day/night cycles. The core mechanism of the mammalian clock consists of heteromeric transcription complex BMAL1:CLOCK that transcribes cryptochrome (*Cry1* & *Cry2*) and period (*Per1*, *Per2*, and *Per3*) genes. CRYs and PERs heterodimerize, translocate to the nucleus, repress BMAL1:CLOCK transcriptional activity and undergo proteasomal degradation. This transcription-translation feedback loop takes about 24 h. In addition, BMAL1:CLOCK drive expression of REV-ERBα/β that inhibit transcription of *Bmal1*.

As is seen in Figure 1C, expression of *Bmal1* and *Per1* oscillates in synchronized cultured adipocytes in a cell autonomous fashion, and their phases are, as expected, reverse. Expression of Egr1 also demonstrates self-sustained circadian pattern in synchronized cultured 3T3-L1 adipocytes (Figure 1D). Noteworthy, circadian phases of *Bmal1* and *Per1,* are different from that of *Egr1*. Clearly, there should be other factors that are responsible for the circadian rhythm of the *Egr1* expression. To this end, various actinomycin D- and *α*-Amanitin-resistant (i.e., *non-transcriptional*) circadian clocks have been described by many research groups in the course of the years [reviewed in (Reddy and Rey, 2014)].

We have found that oscillations in the Egr1 mRNA and protein in synchronized adipocytes correlate with rhythmic changes in the activity of the MEK/Erk and mTORC1 pathways (Figure 1E). Indeed, there are distinct peaks in phosphorylation of MEK, Erk, and S6K1 at 6, 18, and 36–42 h that overlap with or slightly precede peaks of the Egr1 mRNA and protein. Since both MEK/Erk & mTORC1 pathways directly control expression of Egr1, a close correlation between these events may prove to have a causative connection. Furthermore, oscillations of the MEK/Erk and/or mTORC1 pathways may represent a totally novel type of an endogenous circadian regulator or may be linked to the autonomous cycling of the known “core” clock genes *via* an as yet unknown mechanism. In any case, understanding their molecular nature seems warranted.

Strong evidence supports the idea that BMAL1 contributes to the circadian pattern of the ATGL (Shostak et al., 2013b) and leptin (Paschos et al., 2012) expression in adipocytes. An interesting question is whether and to what extent Egr1 can also regulate insulin-independent circadian expression of its direct transcriptional targets, ATGL and leptin. Oscillation patterns of either leptin or ATGL mRNA do not apparently overlap with cycling of *Egr1* or *Bmal1*. This is to be expected as both leptin and ATGL promoters are regulated by various transcription factors with their own cycling patterns, so the resulting picture may be complex. Most genes in various tissues demonstrate the same phenomenon (Fang et al., 2014; Shostak and Brunner, 2019), and its biological sense is not completely understood.

Importantly, both FA and leptin represent signaling molecules that work on hypothalamic neurons to regulate physiological rhythms of the whole organism (Ahima and Flier, 2000; Lam et al., 2005). Therefore, further studies of the cell autonomous biological clock in adipocytes that regulate expression of FA and leptin should have a global physiological significance.

## Discussion

As is pointed out in the previous section, both lipolysis and food intake are regulated by nutrients/insulin as well as by endogenous circadian rhythms. It is essential to inhibit lipolysis at the time of food abundance and to activate lipolysis upon fasting and to coordinate these responses with endogenous circadian rhythm that adapts the organism to cyclic changes of the environment.

Disruption of insulin (McGarry, 2002) and circadian (Shostak et al., 2013a; Froy and Garaulet, 2018; Kolbe et al., 2018; Lemmer and Oster, 2018; Pilorz et al., 2018) regulation of lipolysis and food intake is associated with obesity, insulin resistance, and metabolic diseases. Although a temporal misalignment of feeding time and circadian rhythms may be metabolically acceptable, there is no question that systemic ignoring and abuse of circadian rhythms disrupts metabolic homeostasis (Cederroth et al., 2019). At present, there is little understanding of the interplay between circadian rhythms and metabolic regulation. This question is directly related to human health and thus represents a high priority direction of research (Eckel-Mahan et al., 2013; Asher and Sassone-Corsi, 2015; Cederroth et al., 2019; Duglan and Lamia, 2019).

**FIGURE 2:**
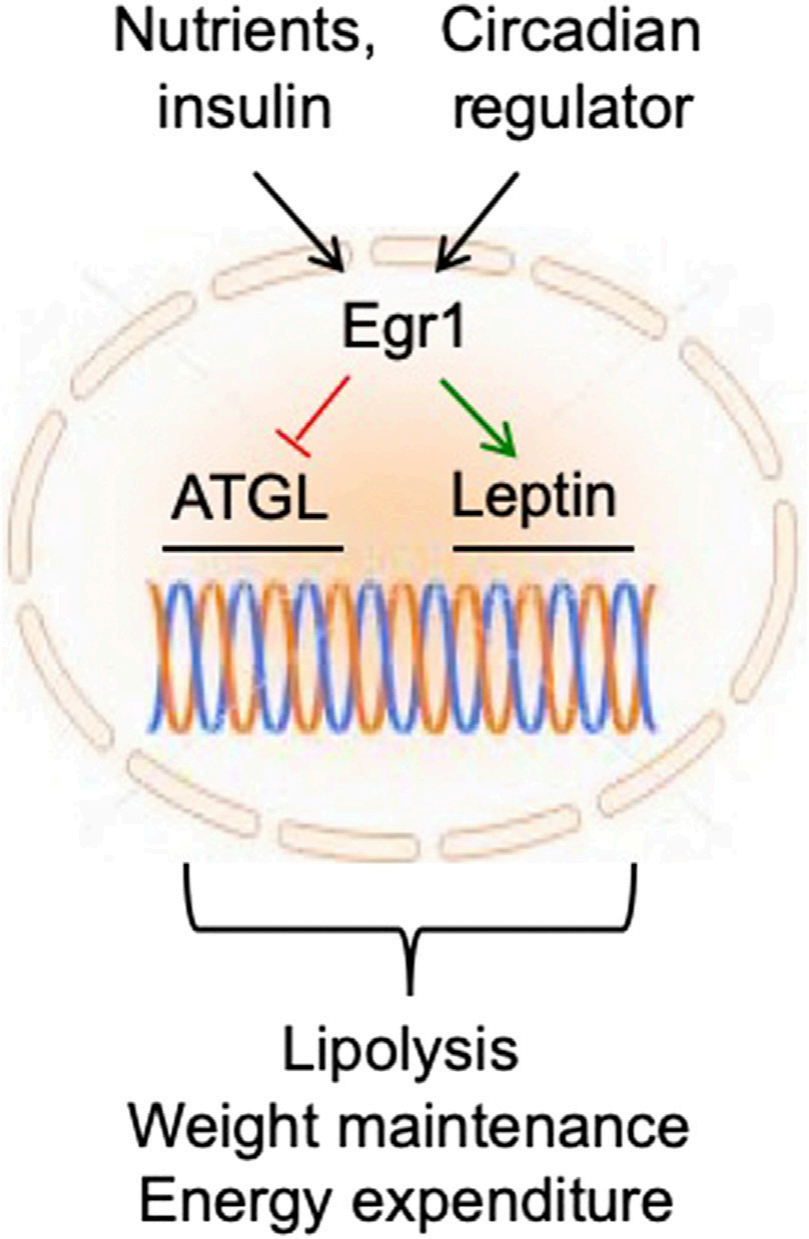
In fat tissue, Egr1 receives regulatory inputs from nutrients/insulin and circadian regulator and maintains metabolic health and longevity by suppressing ATGL and activating leptin expression.

Since Egr1 responds not only to metabolic signals (i.e., nutrients and insulin) but also, to an endogenous circadian pacemaker in adipocytes, it is well suited to coordinate metabolic and circadian regulation of lipolysis, food intake, and energy expenditure (Figure 2). This robust system may have evolved to provide metabolic stability to the organism under unpredictable life conditions. For example, in the past, animals used to live in the same time zone, but food was scarce and its availability was random. Therefore, strong circadian regulation of Egr1 in both nocturnal and diurnal animals could play the primary role in the adjustment of their metabolism to dark/light cycles regardless of food supply.

In modern humans the situation is reversed. Counter to our diurnal nature, we often work night shifts or travel through multiple time zones. At the same time, food has become more available, and we can take advantage of time-restricted feeding to correct negative metabolic consequences of disordered molecular clock (Vollmers et al., 2009; Hatori et al., 2012; Sherman et al., 2012; Chaix et al., 2014). Thus, dysregulation of the circadian pattern of Egr1 expression may be compensated by strengthening the nutrient-based regulatory axis (in particular, by time-restricted feeding) and *vice versa*.

## Data Availability

The original contributions presented in the study are included in the article/Supplementary Materials, further inquiries can be directed to the corresponding author.

## Supplementary material

SUPPLEMENTARY FIGURE S1 Incubation of cultured 3T3-L1 adipocytes with glucose increases expression of Egr1. Panel **(A)**: Cells were incubated in the glucose-free medium for 2.5 h, then glucose (20 mM) or insulin (100 nM) were added for 1.5 h and the Egr1 mRNA was measured by RT-qPCR. Panel **(B)**: Cells were incubated in the glucose-free medium for 6 h, then glucose (Glu, 20 mM) or insulin (Ins, 100 nM) were added for the indicated time, and total cell extracts were analyzed by Western blotting. Dotted lines indicate that irrelevant bands have been spliced out. The experiment was repeated twice. The following antibodies from Cell Signaling Technology have been used: rabbit monoclonal antibody against Egr1 (Catalog #4153), mouse monoclonal antibody against *β*-Actin (Catalog #3700).

SUPPLEMENTARY FIGURE S2 Insulin does not stimulate degradation of Egr1. 3T3-L1 adipocytes were labeled with a mixture of [^35^S]-labeled cysteine and methionine for 1 h, after which the cells were harvested or chased with 2 mM of unlabeled amino acids in the absence and in the presence of 100 nM insulin for the indicated amounts of time. Panel **(A)**: Egr1 was immunoprecipitated from cell lysates using rabbit monoclonal antibody against Egr1 (Cell Signaling Technology, Catalog #4153) or normal rabbit IgG (Cell Signaling Technology, Catalog #2729) (CON) and analyzed by SDS electrophoresis and autoradiography. Panel **(B)**: The membrane shown in Panel **(A)** was further blotted with HRP-linked secondary anti-rabbit IgG (Cell Signaling Technology, Catalog #7074) for the IgG Heavy Chain to confirm that equal amounts of the anti-Egr1 antibodies were used in the experiment. Panel **(C)**: Prior to immunoprecipitation, total cell extracts (TE) were analyzed by SDS electrophoresis and autoradiography.

SUPPLEMENTARY FIGURE S3 The inhibitory effects of insulin and Actinomycin D on ATGL transcription in cultured 3T3-L1 adipocytes. Differentiated 3T3-L1 adipocytes were incubated with or without insulin (100 nM) and Actinomycin D (5 μM) for the indicated amounts of time. Panel **(A)**: *Atgl* mRNA was measured by RT-qPCR in three independent experiments; mean values +/− SE are shown. Panel **(B)**: Total cell extracts were analyzed by Western blotting with rabbit polyclonal antibody against ATGL (Cell Signaling Technology, Catalog #2138) and mouse monoclonal antibody against *β*-Actin (Cell Signaling Technology, Catalog #3700).
