# Supplementary figures and images for: Egr1 plays a major role in the transcriptional response of white adipocytes to insulin and environmental cues
Source: Front Cell Dev Biol. 2022 Sep 28;10:1003030. doi: 10.3389/fcell.2022.1003030 (PMC9554007; doi:10.3389/fcell.2022.1003030)

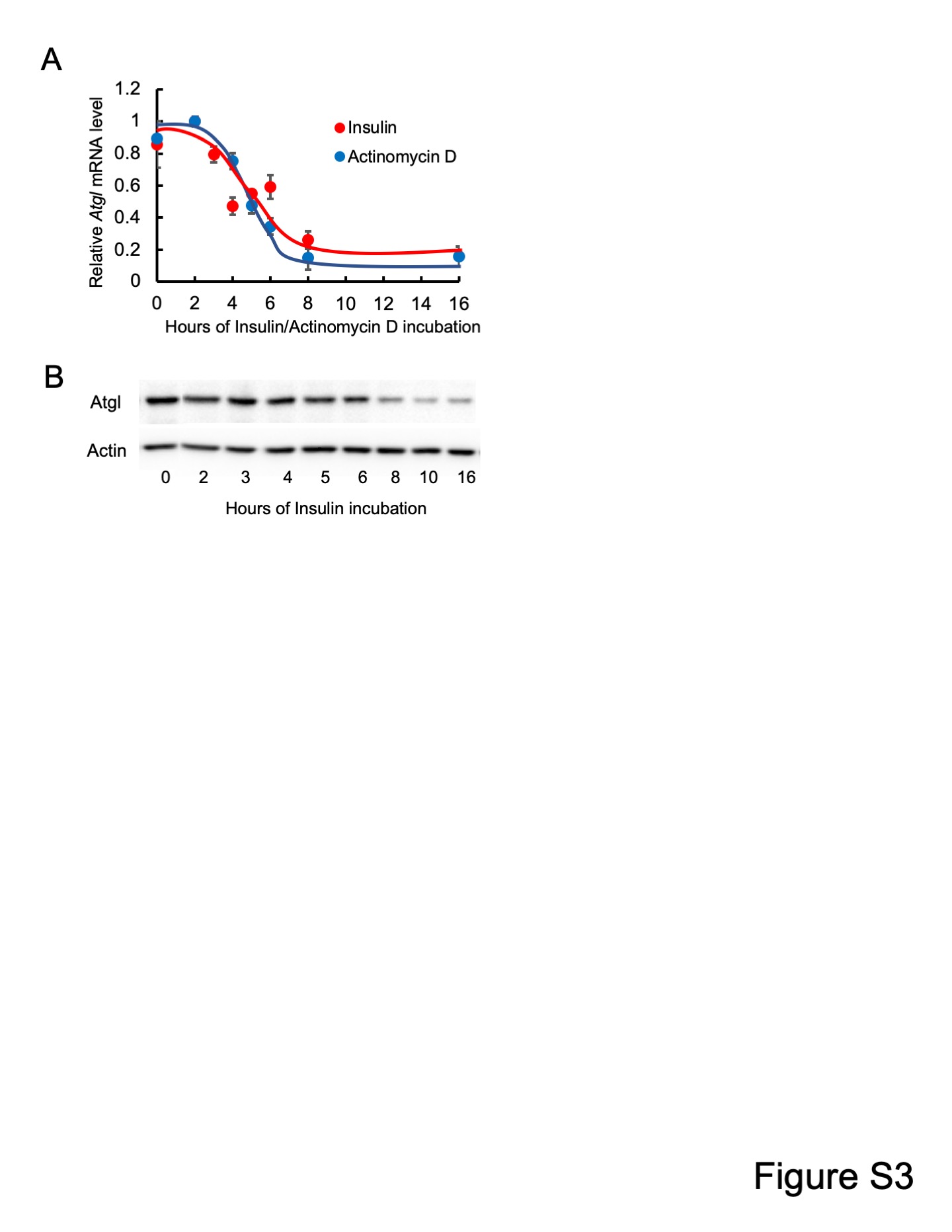

Supplement: Supplementary file 1 [file Image3.jpeg]

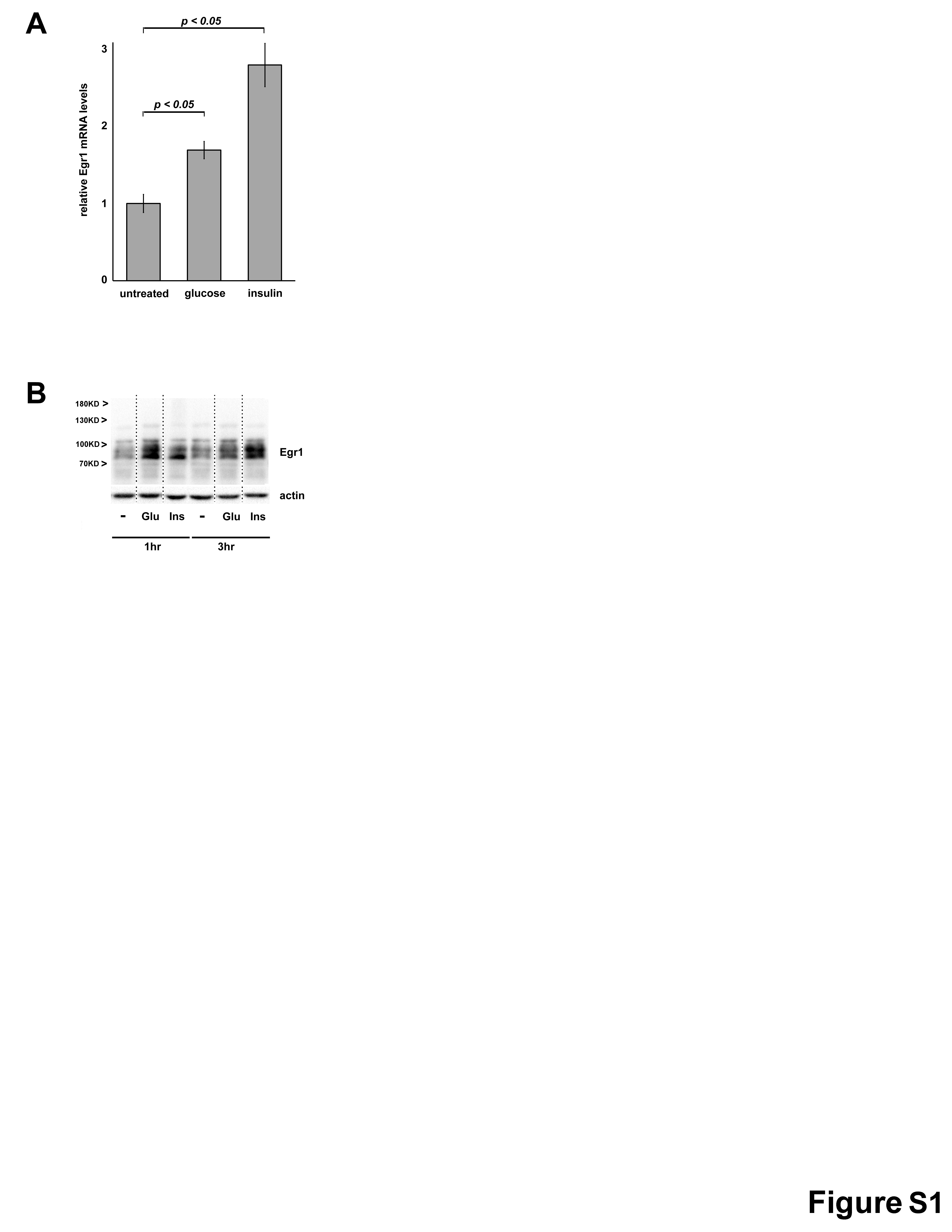

Supplement: Supplementary file 2 [file Image1.jpeg]

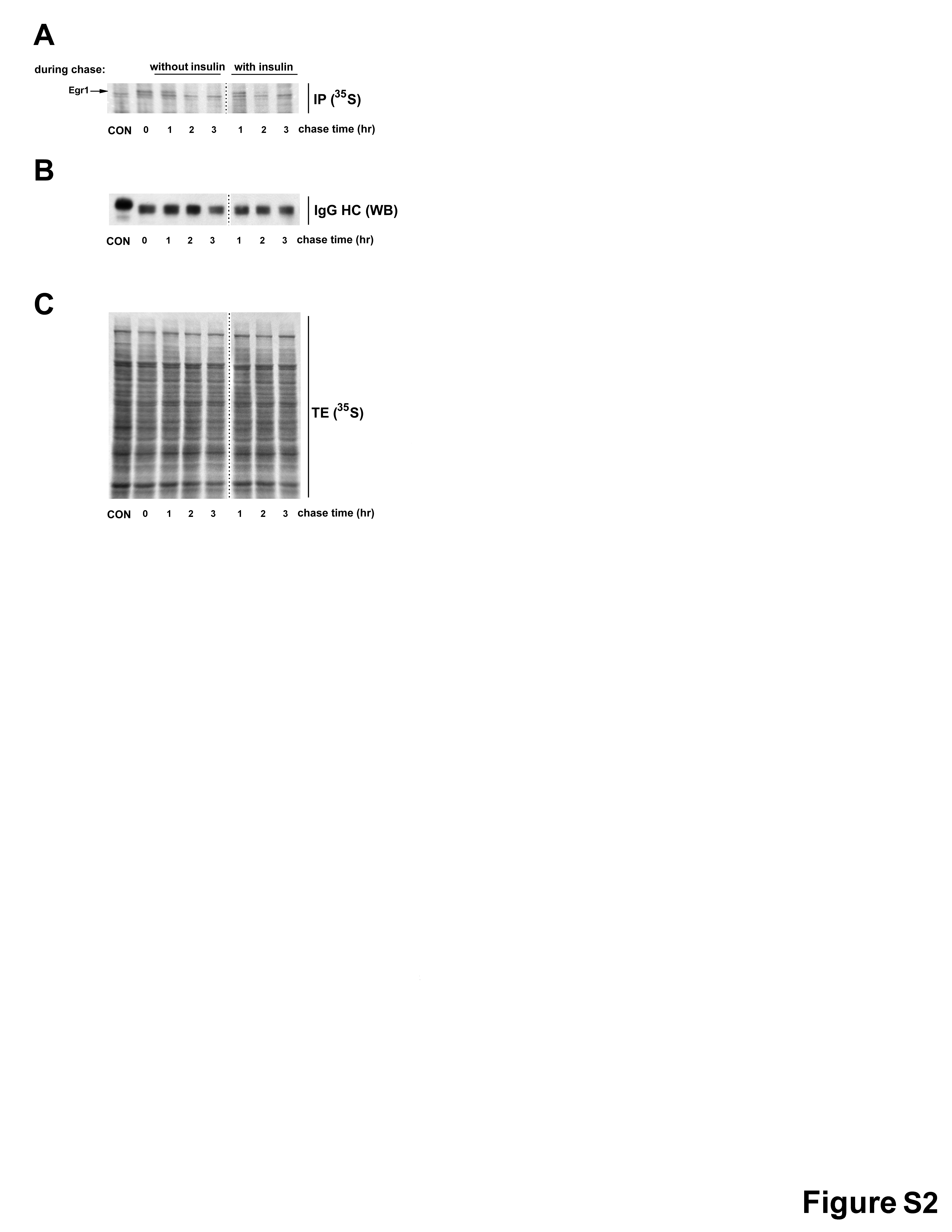

Supplement: Supplementary file 3 [file Image2.jpeg]
